# A Fishy Situation: Hand Infection due to *Mycobacterium marinum* Mistaken for Giant Cell Tumor

**DOI:** 10.1155/crdi/8021905

**Published:** 2026-01-29

**Authors:** Alynna Knaub, Dylan Baker, Jennifer Hanrahan

**Affiliations:** ^1^ Department of Medicine, Macon and Joan Brock Virginia Health Sciences at Old Dominion University, Norfolk, Virginia, USA, odu.edu; ^2^ Department of Flight Medicine, Tyndall Air Force Base, Panama City, Florida, USA; ^3^ Department of Internal Medicine, Marshall Health Network, Huntington, West Virginia, USA

## Abstract

*Mycobacterium marinum* is an acid‐fast bacterium (AFB) associated with exposure to water and aquatic species. When inoculated, infection can result in nodular cutaneous lesions. In the absence of detailed history or culture data, these nodular skin lesions can be mistaken for noninfectious orthopedic conditions. We present a case of *M. marinum* mistaken for a giant cell tumor. This case illustrates the overlap in these conditions, as well as the utility of QuantiFERON Gold testing to provide supportive evidence for the diagnosis of *Mycobacterium marinum*.

## 1. Introduction

*M. marinum*​ is a pathogen associated with exposure to contaminated water or aquatic species. The incubation period can be up to 9 months [1], causing delayed clinical presentation and key details of exposure to be forgotten. Given its low incidence (approximately 0.00027% annually in the United States [2]), healthcare providers may not be familiar with the presentation and treatment of this problem. Infection can present in a variety of ways but most commonly presents as cutaneous disease with a solitary nodular lesion on a finger or hand. On occasion, cutaneous lesions caused by *M. marinum* become invasive and have been mistakenly treated as noninfectious conditions such as degenerative tenosynovitis [2]. Differential diagnosis should include other mycobacterial infections, fungal infections, granulomatous infections, and bone neoplasms. *M. marinum* can be diagnosed via positive cultures, most commonly from blood and tissue [2]. The presence of granulomas can support a diagnosis of *M. marinum,* but has low sensitivity [2]. When there is delay in diagnosis and antibiotic treatment, surgical debridement may be required for source control [1–3]. Though intended to diagnose latent *Mycobacterium tuberculosis* infection [4], QuantiFERON Gold testing can be used as a supportive diagnostic tool because it detects the immune response to specific proteins (ESAT‐6 and CFP‐10) found in both *M*. *tuberculosis* and *M. marinum*.

## 2. Case Presentation

**FIGURE 1:**
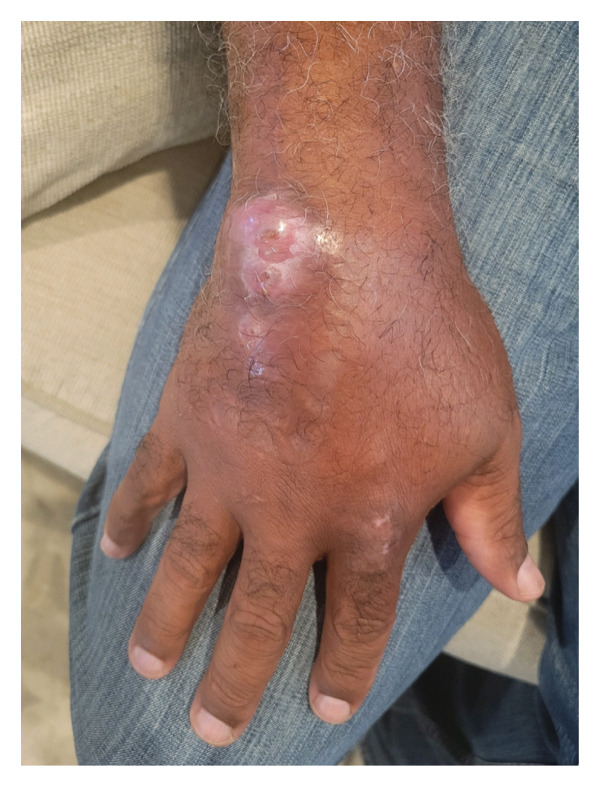
Nodular lesion from *Mycobacterium marinum* at the initiation of antibiotic treatment.

**FIGURE 2:**
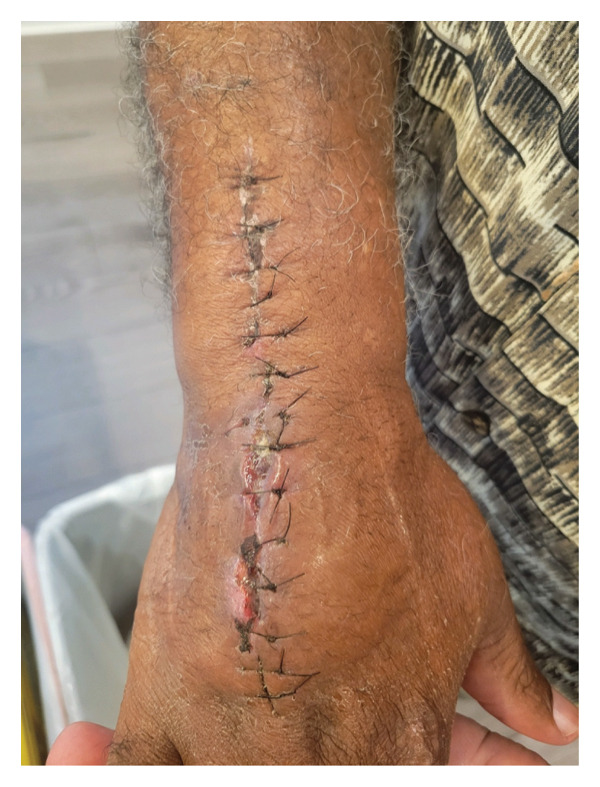
*Mycobacterium marinum* hand lesion following second surgical debridement.

A 62‐year‐old man with a history of hypertension presented to an orthopedic surgeon with a nodular lesion on his hand (Figure 1). He cut his hand months prior while shelling shrimp, and the lesion was mistaken for a wart and removed several times. He developed hand and forearm swelling and underwent resection for a presumed giant cell tumor. Pathology demonstrated necrotizing granulomas. The patient was referred to the infectious disease department for evaluation. Due to inconclusive surgical pathology and appearance of nodular lymphangitis, as well as the patient’s exposure to shellfish, a QuantiFERON Gold test was ordered to rule out *M. marinum.* The QuantiFERON Gold test was positive, and azithromycin and ethambutol were initiated for presumed *M. marinum.* Due to the expansion of the nodular lesion, repeat surgical debridement was performed. Intraoperative wound culture was positive for *M. marinum*. Following surgical debridement and completion of a 12‐month course of antibiotic therapy, the lesion resolved (Figure 2).

## 3. Discussion

When treating nonhealing nodular skin lesions, a careful history should include exposure to water, shellfish, fish tanks, or boating. *M. marinum* infection is introduced via an aquatic medium, and cutaneous manifestations are commonly referred to as “fish tank granulomas” [5]. It is an often overlooked and misdiagnosed cause of nonhealing skin lesions, with a long incubation period [1].

Treatment of orthopedic conditions with steroid injections can worsen *M. marinum* infection [2]. Deep infections, either from direct inoculation or disease progression, can result in infectious tenosynovitis, osteomyelitis, arthritis, and bursitis, which occur in 20%–40% of cases [5]. Systemic dissemination is also possible but has only been reported in immunocompromised patients. Localized adenopathy is infrequent and occurs in approximately 15% of cases [5]. When the infection spreads to deeper structures, surgical debridement is often needed [2], as in our case.

Histology is not a sensitive method of diagnosis, as granulomas may not be present and acid‐fast stains may be negative [2]. Even if detected, granulomas, positive acid‐fast bacteria (AFB) staining, and giant cells are not unique to this pathogen [2, 6]. Cultures remain the gold standard for diagnosis, though they have been noted to be negative in nearly 50% of cases [7]. Cultures also require an adequate specimen sample and an incubation period ranging from one to several weeks [5].

The QuantiFERON‐TB Gold test has cross‐reactivity with *M. marinum, M. kansasii,* and *M. tuberculosis* [8]. QuantiFERON Gold testing can aid in providing supportive evidence of *M. marinum* in those with low probability for latent tuberculosis. A positive QuantiFERON Gold test, physical exam findings, and a history of potential support the clinical diagnosis of *M. marinum*.

*Mycobacterium marinum* and giant cell tumors both present as nodular lesions and have multinucleated giant cells on pathologic examination [7, 9] and can be mistaken for each other. To our knowledge, this overlap has not been previously reported in the literature. In individuals with low likelihood of tuberculosis, a positive QuantiFERON Gold assay can serve as a supportive tool for the diagnosis of *M. marinum* if initial culture or pathologic data are inconclusive.

Testing for *M. marinum* with AFB cultures should be obtained when possible. The QuantiFERON Gold assay may provide supportive evidence in individuals who are unlikely to have exposure to tuberculosis, though culture remains the gold standard. Nontuberculous mycobacterial infection should be considered in any refractory skin lesions, especially in those exposed to aquatic environments [1, 2].

## Funding

The authors received no specific funding for this work.

## Consent

Verbal patient consent was obtained via phone by Dr. Hanrahan. No written consent has been obtained from the patient as there are no patient identifiable data included in this case report.

## Conflicts of Interest

The authors declare no conflicts of interest.
